# Impact of Sleeve Gastrectomy on Psychiatric Medication Use and Symptoms

**DOI:** 10.1155/2018/8532602

**Published:** 2018-10-15

**Authors:** Scott V. Monte, Kristen M. Russo, Esra Mustafa, Joseph A. Caruana

**Affiliations:** ^1^University at Buffalo School of Pharmacy and Pharmaceutical Sciences, 213 Kapoor Hall, Buffalo, NY 14214, USA; ^2^Synergy Bariatrics, A Department of ECMC, University at Buffalo School of Medicine, Buffalo, NY 14214, USA

## Abstract

**Background:**

Sleeve gastrectomy (SG) has become the primary bariatric surgery procedure in the U.S. Over 50% of people presenting for surgery have psychiatric diagnoses. The study purpose was to evaluate change in anxiety and depression symptoms and medication use after SG.

**Methods:**

Subjects completing SG with a diagnosis of anxiety and/or depression treated with medication were retrospectively identified from the electronic medical record (EMR) of Synergy Bariatrics, a department of the Erie County Medical Center. Phone outreach was made to complete seven-point global impression of change scale classifying symptom improvement or worsening in the 3- to 6-month postoperative period. Improvement or worsening was defined as either all reported symptoms improving or worsening or ≥1 improving or worsening while remaining unchanged. If ≥1 symptom improved and worsened, it was classified as mixed. No change required the same profile before and after surgery. Medication, dose, and changes were identified by EMR, verified during interview and classified as no change, discontinued, decreased, or increased.

**Results:**

59 subjects completed the interview. 21 subjects were diagnosed with anxiety. 13 (62%) had no change in therapy and 5 (24%) decreased. Symptoms improved in 12 (57%), worsened in 3 (14%), and mixed in 5 (24%). When symptoms improved, the same dose was present in 7/12 (58%) and dose decreased in 3 (25%). 51 subjects were diagnosed with depression. 32 (63%) had no change in therapy, 11 (21%) discontinued, and 4 (8%) decreased. Symptoms improved in 34 (67%), mixed in 10 (20%), worsened in 4 (8%), and unchanged in 3 (6%). When symptoms improved, the same regimen and dose was present in 21/34 cases (62%) and discontinued in 9 (26%).

**Conclusion:**

Anxiety symptoms improved in >50% of subjects at predominantly the same or reduced dosage. Depression symptoms improved in 67% and commonly without therapy change. These data suggest evidence that patients undergoing SG while on medication for anxiety or depression may have early symptom improvement on the same or lessened dosage.

## 1. Introduction

The National Institutes of Health estimates that 36% of adults in the United States are obese [1]. Bariatric surgery procedures Roux-en-Y gastric bypass (RYGB) and Sleeve gastrectomy (SG) are well-established interventions for weight loss [2]. RYGB was the most common procedure performed until 2014 when SG increased to >50% of cases [3]. One advantage of SG in comparison with RYGB is thought to be lessened concern for altered medication absorption given that only a portion of the stomach is removed and the transition to the small intestine remains continuous. This is of particular importance to people using medication therapy for depression and anxiety.

People presenting for bariatric surgery are diagnosed with depression in 36% of cases and anxiety in 11% [4]. Cunningham et al. found that less than half of people remain on the same antidepressant regimen after RYGB. In approximately 40% of those cases, an increase or switch in antidepressant was required. Only 15% decreased or discontinued therapy [5]. These data are consistent with reports of reduced absorption of selective serotonin reuptake inhibitors (SSRIs) and tricyclic antidepressants after RYGB [6]. Antianxiety medication use and absorption is not widely studied after RYGB. Prescription fill data suggest an increased use of antianxiety, sedative, and hypnotic medications following RYGB [4]. In comparison with RYGB, considerably less is known about depression and anxiety symptoms and medication use after SG. Mack et al. identified improvement in depression and stress symptoms in the 48-month period after SG [7]. However, there are no antidepressant or antianxiety medication absorption and efficacy studies after SG [8]. No studies have characterized or evaluated the overlap of change in depression or anxiety symptoms and change in medication use after RYGB or SG.

The objectives of this study were to characterize depression and anxiety symptom change; characterize antidepressant and antianxiety medication use change; and identify predictors of symptom improvement or worsening after SG.

## 2. Materials and Methods

Subjects with a diagnosis of depression, anxiety, or both treated with antidepressant or antianxiety medication at the time of SG surgery were identified from medical records of Synergy Bariatrics, a department of the Erie County Medical Center (Buffalo, NY) in 2016. Study investigators contacted patients by phone, and following informed consent, they were interviewed to determine change in depression and anxiety symptoms and medication use in the 3- to 6-month period after surgery. At the time of the interview study, investigators reviewed the medical chart to verify medication names, doses, and changes in therapy with the subject. Subjects were excluded if they became pregnant or experienced rehospitalization related to surgery, reoperation, or had another major surgery in the 3- to 6-month evaluation period.

A hard copy of the seven-point patient global impression of change scale (PGIC) was used to conduct and document subject survey responses (see Supplementary Appendix 1). The Patient Global Impression of Change (PGIC) is a self-report measure that reflects a patient's belief about the efficacy of treatment. PGIC is a seven-point scale that depicts a patient's rating of overall improvement [9]. Depression symptoms evaluated included feeling down or hopeless, little interest or pleasure in doing things, trouble falling asleep, sleeping too much, feeling tired or having little energy, trouble concentrating, overeating, self-dislike, and suicidal thoughts. Anxiety symptoms evaluated were anxious mood, feeling tense, feeling restless, crying easily, trouble falling asleep, trouble concentrating, little interest or pleasure in doing things, feeling tired or having little energy, and being afraid of the dark, strangers, being alone, or crowds. Investigators assessed both depression and anxiety symptoms in patients with both diagnoses. Patients with both diagnoses were treated with medications that are commonly used to treat both depression and anxiety. Symptom changes were designated as very much worse, much worse, minimally worse, minimally improved, much improved, very much improved, and those having no change versus baseline. Symptom improvement was defined as either all symptoms improving or at least one symptom improving while the remaining were unchanged. The same criteria were applied for symptom worsening. A mixed profile was noted if at least one symptom improved and worsened. No change required the same profile before and after.

Medication use was categorized as unchanged, increased, decreased, discontinued, or a switch in medication class. Increase in medication use was defined as either an increase in medication dose or frequency. The same criteria were applied for decrease in medication use.

The medical record review supplemented the interview to gather patient demographics, body mass index (BMI), excess body weight, ideal body weight, percentage of body weight lost and psychiatric diagnoses, medications, and doses. All data were maintained in a Microsoft Excel file on the internal servers of the medical practice. Data are presented descriptively and in the tabular form for change in depression and anxiety symptoms, change in medication use, and combined symptom change and medication use. Simple logistic regression was used to identify associations between independent variables and symptom improvement. Independent variables included age, gender, height, ideal body weight, weight, and excess body weight at the time of surgery, and excess body weight and percentage of excess body weight loss after surgery, death in the family, marriage or divorce, pregnancy, new or lost job, emergency room visit or hospitalization unrelated to surgery, transfer addictions of new or increased alcohol or tobacco use, prescription or recreational drug abuse, and eating disorders. Transfer addiction, after bariatric surgery, occurs when individuals trade compulsive eating for other compulsive behaviors. Other behaviors or substances may substitute for eating.

The protocol for the study was approved by the State University of New York at Buffalo Institutional Review Board.

## 3. Results and Discussion

Fifty-nine subjects completed interview: 38 with depression, eight with anxiety, and 13 with depression and anxiety. Subject characteristics are shown in Table 1.

### 3.1. Depression

Change in depression symptoms and medication use for 51 subjects are shown in Table 2. Depression symptoms improved in 34/51 (67%) subjects in the 3–6 months following SG.

Age, gender, height, ideal body weight, weight and excess body weight at the time of surgery, and percentage of excess body weight loss after surgery were not significantly associated with symptom change after SG (*p* > 0.1 for all). Death in the family, marriage, divorce, new job, lost job, and transfer addiction were also not significantly associated with symptom change. Medication use changes (no change in medication, medication increase, medication decrease, medication discontinuation, or switch in medication class) were not significantly associated with symptom change after SG. In cases where symptoms improved, 32 (94%) occurred in circumstances where medications were discontinued, decreased, or unchanged. Symptoms worsened in four (8%), were unchanged in three (6%), and were mixed in 10 (20%).

### 3.2. Anxiety

Change in anxiety symptoms and medication use for 21 subjects are shown in Table 3. Anxiety symptoms improved in 12/21 (57%) subjects. Gender, height, ideal body weight, weight and excess body weight at the time of surgery, and percentage of excess body weight loss after surgery were not significantly associated with symptom change after SG (*p* > 0.1 for all). Increased age was predictive of symptom improvement (*p*=0.041). Death in the family, marriage, divorce, new job, and transfer addiction were not significantly associated with symptom change. No patients lost job. Medication use changes (no change, medication increase, medication decrease, or medication discontinuation) were not significantly associated with symptom change after SG. No patients changed medication class. In cases where symptoms improved, 11 (92%) occurred in circumstances where medications were discontinued, decreased, or unchanged. Symptoms worsened in three (14%), were unchanged in one (5%), and were mixed in five (24%).

This study is the first to characterize depression and anxiety symptom change and antidepressant and antianxiety medication use change and evaluate predictors of symptom improvement or worsening after SG. We have shown that a high percentage of patients experience improvement in their depression (67%) and anxiety (57%) symptoms after SG. Intuitive factors expected to be predictive of symptom improvement such as excess body weight at the time of surgery and percentage of excess body weight lost after surgery were not significantly associated with depression or anxiety symptom improvement. In fact, age was the only predictor of symptom improvement and observed in only the anxiety group. Strikingly, 94% and 92% of depression and anxiety subjects experienced symptom improvement while at the same time discontinuing, decreasing, or not changing their medication, dose, or formulation. Taken together with the lack of other independent predictors for symptom improvement, these data may suggest that SG has an independent effect on depression and anxiety. This finding would be consistent with the known effect of SG to reduce inflammatory cytokines such as interleukin-6 and C-reactive protein that are heightened in depression and anxiety [10, 11].

Antidepressant and antianxiety medication change should be anticipated in the first 3–6 months after SG. The antidepressant regimen was altered in 37% of cases. The majority discontinued or decreased medication, but four subjects (19%) required a dose increase or class switch. Antianxiety data were similar to depression in that the regimen was altered in 38% of cases and the majority discontinued or decreased medication. Two subjects required dose increase (9%). These cases where medication was increased or class switched leave open the possibility of antianxiety or antidepressant absorption being compromised in a minority of people after SG.

These data are informative to bariatric surgery providers evaluating and preparing approximately 33% people with depression and 10% with anxiety presenting for bariatric surgery but should be interpreted in the context of the following limitations. First, the sample size is small in 59 subjects. This is a retrospective study with no control group for comparison. Secondly, the evaluation period of 3–6 months after surgery affords no insight into sustainability of symptom improvement. This study examines only the preliminary stages of mental and emotional adjustment to SG and weight loss and provides no opportunity for long-term follow-up. Thirdly, antidepressant use was predominantly SSRI (66%) and serotonin norepinephrine reuptake inhibitor (29%) and antianxiety use was predominantly benzodiazepine (22%). Lastly, the investigators did not inquire about alternative explanations for symptom improvement such as initial weight loss or sense of relief due to a successful surgery.

## 4. Conclusion

Depression and anxiety symptoms improved in a majority of cases after SG. Antidepressant and antianxiety medication regimens were altered in approximately one-third of cases but were primarily reflected by dose decrease or discontinuation. Approximately 10–20% of cases were dose increases or class switches leaving open the possibility of compromised absorption. In depression and anxiety cases where symptoms improved, the medications were decreased or discontinued in over 90%. In the noted absence of clinical and medication factors predicting symptom improvement, these data may suggest an independent effect of SG on depression.

## Figures and Tables

**Table 1:** Subject characteristics (*n*=59).

Patient demographics (*n*=59)	
Age	51 ± 13 years
Gender	51 females (86%)
Height	165 ± 8 cm
Weight	129 ± 28 kg
BMI	46.9 ± 8.2 kg/m^2^
Ideal body weight	58 ± 9 kg
Excess body weight	71 ± 25 kg

Medication classes	No. of patients

SSRI	39 (66%)
SNRI	17 (29%)
Benzodiazepine	13 (22%)
Anticonvulsant	8 (14%)
Atypical antipsychotic	4 (7%)
Bupropion	4 (7%)
Antihistamine	1 (2%)
Trazodone	1 (2%)

BMI = body mass index; SSRI = selective serotonin reuptake inhibitor; SNRI = serotonin norepinephrine reuptake inhibitor.

**Table 2:** Change in depression symptoms compared to change in medication use (*n*=51).

Change in depression symptoms					
Symptom change		Number of patients (*n*=51)		Percentage of patients	

Unchanged		3		6%	
Improved		34		67%	
Worsened		4		8%	
Mixed		10		19%	

Change in medication use					
Medication change		Number of patients (*n*=51)		Percentage of patients	

Unchanged		32		63%	
Increased		2		4%	
Decreased		4		8%	
Discontinued		11		21%	
Switch class		2		4%	

Change in symptoms vs. change in medication use					
Symptom change	Med unchanged	Med increased	Med decreased	Med discontinued	Switch med class

Unchanged	2 (4%)	0 (0%)	1 (2%)	0 (0%)	0 (0%)
Improved	21 (41%)	1 (2%)	2 (4%)	9 (17%)	1 (2%)
Worsened	1 (2%)	1 (2%)	0 (0%)	1 (2%)	1 (2%)
Mixed	8 (16%)	0 (0%)	1 (2%)	1 (2%)	0 (0%)

**Table 3:** Change in anxiety symptoms compared to change in medication use (*n*=21).

Change in anxiety symptoms					
Symptom change		Number of patients (*n*=21)		Percentage of patients	

Unchanged		1		5%	
Improved		12		57%	
Worsened		3		14%	
Mixed		5		24%	

Change in medication use					
Medication change		Number of patients (*n*=21)		Percentage of patients	

Unchanged		13		62%	
Increased		2		9%	
Decreased		5		24%	
Discontinued		1		5%	
Switch class		0		0%	

Change in symptoms vs. change in medication use					
Symptom change	Med unchanged	Med increased	Med decreased	Med discontinued	Switch med class

Unchanged	0 (0%)	0 (0%)	1 (5%)	0 (0%)	0 (0%)
Improved	7 (33%)	1 (5%)	3 (14%)	1 (5%)	0 (0%)
Worsened	2 (9%)	1 (5%)	0 (0%)	0 (0%)	0 (0%)
Mixed	4 (19%)	0 (0%)	1 (5%)	0 (0%)	0 (0%)

## Data Availability

The data used to support the findings of this study are included within the article.

## Supplementary Materials

Supplementary Materials The supplementary material file contains the survey questionnaire used by the study investigators to interview subjects.
