# Supplementary material for: Impact of Sleeve Gastrectomy on Psychiatric Medication Use and Symptoms
Source: J Obes. 2018 Oct 15;2018:8532602. doi: 10.1155/2018/8532602 (PMC6205308; doi:10.1155/2018/8532602)
Supplement: Supplementary Materials — The supplementary material file contains the survey questionnaire used by the study investigators to interview subjects. [file 8532602.f1.docx]

**Patient Survey**

**Change in Anxiety and Depression Symptoms and Medication Use After Sleeve Gastrectomy**

**Patient Name**: _____________________________________________ **DOB** ____/______/______

**Gender**: M / F

**Sleeve Gastrectomy Surgery Date**: ____/______/_______

**Verification of Exclusion Criteria (all must be no to proceed):**

- Did you have a leak after surgery: Y / N
- Did you have a wound infection requiring hospitalization after surgery? Y / N
- Did you have any other major surgeries in the first 3-6 months after surgery? Y / N
- Previous adjustable gastric band or gastric bypass? Y / N

**BMI at Surgery**: _______ kg/m^2^; **IBW at Surgery: _____ (lbs) EBW at Surgery ______ (lbs)**

**Verification of Inclusion Criteria (at least one diagnosis AND medication required to proceed):**

**Anxiety and/or Depression Diagnoses:**

- Anxiety
- Depression
- None

**Anxiety and/or Depression medications at time of Sleeve Gastrectomy (indicate name, dose, directions, indication, ER):**

- Benzodiazepine: ___________________________________________________________
- Bupropion: ________________________________________________________________
- Buspirone: ________________________________________________________________
- Mirtazapine: ______________________________________________________________
- SSRI: _____________________________________________________________________
- SNRI: _____________________________________________________________________
- TCA: _____________________________________________________________________
- MAOI: ____________________________________________________________________
- Mixed 5HT-3 agents: _________________________________________________________
- First Generation Antipsychotics: ________________________________________________
- Atypical Antipsychotic: _______________________________________________________
- Antihistamine: ______________________________________________________________
- Anticonvulsant: _____________________________________________________________
- Lithium Salts: _______________________________________________________________
- Other: _____________________________________________________________________

**Did you have any of the following take place within 3-6 months after surgery?**

- Death in the family: ___________________________________________________________
- Marriage: ___________________________________________________________________
- Divorce: ____________________________________________________________________
- Pregnancy: __________________________________________________________________
- New job: ____________________________________________________________________
- Lost job: ____________________________________________________________________
- Emergency room visit (indication): _______________________________________________
- Hospital stay (indication, duration): ______________________________________________
- Diagnosed with a transfer addiction: _____________________________________________
  - New or increased alcohol use
  - New or increased tobacco use
  - New or increased recreational drug use
  - New prescription drug abuse
  - Eating disorder

**Excess Body Weight Loss 3-6 months after surgery (verify from medical record)**

| Surgery Weight: _______ | 6-weeks | 3-months | 6-months |
| --- | --- | --- | --- |
| Weight |  |  |  |
| IBW |  |  |  |
| EBW |  |  |  |
| EBWL |  |  |  |

**Is the subject treated with an antianxiety or antidepressant medication? If yes, complete the following interview:**

| **Table 3. Patient Reported Impression of Psychiatric Symptoms at 3-6 months after Sleeve Gastrectomy** | | | | | | | | |
| --- | --- | --- | --- | --- | --- | --- | --- | --- |
| **Symptoms** | **N/A** | **Very Much Improved** | **Much Improved** | **Minimally Improved** | **No**  **Change** | **Minimally**  **Worse** | **Much**  **Worse** | **Very Much Worse** |
| **Anxiety** |  |  |  |  |  |  |  |  |
| Anxious mood (worries, anticipation of the worst, irritability) |  |  |  |  |  |  |  |  |
| Feeling tense |  |  |  |  |  |  |  |  |
| Feeling restless |  |  |  |  |  |  |  |  |
| Crying easily |  |  |  |  |  |  |  |  |
| Afraid of the dark, of strangers, of being alone, or of crowds? |  |  |  |  |  |  |  |  |
| Trouble falling asleep |  |  |  |  |  |  |  |  |
| Trouble concentrating |  |  |  |  |  |  |  |  |
| Little interest or pleasure in doing things |  |  |  |  |  |  |  |  |
| Feeling tired or having little energy |  |  |  |  |  |  |  |  |
| **Depression** |  |  |  |  |  |  |  |  |
| Feeling down or hopeless |  |  |  |  |  |  |  |  |
| Little interest or pleasure in doing things |  |  |  |  |  |  |  |  |
| Trouble falling asleep |  |  |  |  |  |  |  |  |
| Sleeping too much |  |  |  |  |  |  |  |  |
| Feeling tired or having little energy |  |  |  |  |  |  |  |  |
| Trouble concentrating |  |  |  |  |  |  |  |  |
| Overeating |  |  |  |  |  |  |  |  |
| Self-dislike |  |  |  |  |  |  |  |  |
| Suicidal thoughts |  |  |  |  |  |  |  |  |

| **Table 5. Medication Changes After Sleeve Gastrectomy** | | | | | | | | |
| --- | --- | --- | --- | --- | --- | --- | --- | --- |
|  | **No Change** | **Dose Increase** | **Dose Decrease** | **Formula Change** | **Med Change in same class** | **Med change New Class** | **Med D/C** | **New Med** |
| **Anxiety** |  |  |  |  |  |  |  |  |
| Benzodiazepines |  |  |  |  |  |  |  |  |
| Buspirone |  |  |  |  |  |  |  |  |
| SSRI |  |  |  |  |  |  |  |  |
| SNRI |  |  |  |  |  |  |  |  |
| TCA |  |  |  |  |  |  |  |  |
| MAOI |  |  |  |  |  |  |  |  |
| Atypical antipsychotics |  |  |  |  |  |  |  |  |
| Antihistamine |  |  |  |  |  |  |  |  |
| Anticonvulsant |  |  |  |  |  |  |  |  |
| **Depression** |  |  |  |  |  |  |  |  |
| SSRI |  |  |  |  |  |  |  |  |
| SNRI |  |  |  |  |  |  |  |  |
| TCA |  |  |  |  |  |  |  |  |
| Bupropion |  |  |  |  |  |  |  |  |
| Mirtazepine |  |  |  |  |  |  |  |  |
| MAOI |  |  |  |  |  |  |  |  |
| Mixed 5HT3 Agents |  |  |  |  |  |  |  |  |
| Second Generation Atypical Antipsychotics |  |  |  |  |  |  |  |  |
| Lithium Salts |  |  |  |  |  |  |  |  |
